# Expression Analysis of α5 Integrin Subunit Reveals Its Upregulation as a Negative Prognostic Biomarker for Glioblastoma

**DOI:** 10.3390/ph14090882

**Published:** 2021-08-30

**Authors:** Nelly Etienne-Selloum, Julien Prades, Diana Bello-Roufai, Mathieu Boone, Henri Sevestre, Stéphanie Trudel, Pascal Caillet, Alexandre Coutte, Christine Desenclos, Jean-Marc Constans, Sophie Martin, Laurence Choulier, Bruno Chauffert, Monique Dontenwill

**Affiliations:** 1 Laboratory of Bioimaging and Pathology, Faculty of Pharmacy, University of Strasbourg, UMR7021 CNRS, 67400 Illkirch, France; sophie.martin@unistra.fr (S.M.); laurence.choulier@unistra.fr (L.C.); 2 Service de Pharmacie, Institut de Cancérologie Strasbourg Europe, 67200 Strasbourg, France; 3 Service d’Oncologie Médicale, CHU Amiens-Picardie, 80000 Amiens, France; ju-prades@hotmail.fr (J.P.); bellodiana@yahoo.fr (D.B.-R.); Boone.Mathieu@chu-amiens.fr (M.B.); chauffert.bruno51@gmail.com (B.C.); 4 Service d’Anatomie et Cytologie Pathologique, CHU Amiens-Picardie, 80000 Amiens, France; Sevestre.Henri@chu-amiens.fr; 5 Laboratoire d’Oncobiologie Moléculaire, CHU Amiens-Picardie, 80000 Amiens, France; trudel.s@chu-toulouse.fr; 6 Service Epidémiologie, Hygiène Hospitalière et Santé Publique, Registre du Cancer de la Somme, CHU Amiens-Picardie, 80000 Amiens, France; pascal.caillet@chu-nantes.fr; 7 Service de Radiothérapie, CHU Amiens-Picardie, 80000 Amiens, France; Coutte.Alexandre@chu-amiens.fr; 8 Service de Neurochirurgie, CHU Amiens-Picardie, 80000 Amiens, France; Desenclos.Christine@chu-amiens.fr; 9 Service de Radiologie et Imagerie Médicale, CHU Amiens-Picardie, 80000 Amiens, France; Constans.Jean-Marc@chu-amiens.fr

**Keywords:** biomarker, glioblastoma, integrin α5, Stupp protocol resistance

## Abstract

Integrin α5β1 was suggested to be involved in glioblastoma (GBM) aggressiveness and treatment resistance through preclinical studies and genomic analysis in patients. However, further protein expression data are still required to confirm this hypothesis. In the present study, we investigated by immunofluorescence the expression of integrin α5 and its prognostic impact in a glioblastoma series of patients scheduled to undergo the Stupp protocol as first-line treatment for GBM. The integrin α5 protein expression level was estimated in each tumor by the mean fluorescence intensity (MFI) and allowed us to identify two subpopulations showing either a high or low expression level. The distribution of patients in both subpopulations was not significantly different according to age, gender, recursive partitioning analysis (RPA) prognostic score, molecular markers or surgical and medical treatment. A high integrin α5 protein expression level was associated with a high risk of recurrence (HR = 1.696, 95% CI 1.031–2.792, *p* = 0.0377) and reduced overall survival (OS), even more significant in patients who completed the Stupp protocol (median OS: 15.6 vs. 22.8 months; HR = 2.324; 95% CI 1.168–4.621, *p* = 0.0162). In multivariate analysis, a high integrin α5 protein expression level was confirmed as an independent prognostic factor in the subpopulation of patients who completed the temozolomide-based first-line treatment for predicting OS over age, extent of surgery, RPA score and O-6-methylguanine-DNA methyltransferase (MGMT) promoter methylation (*p* = 0.029). In summary, for the first time, our study validates that a high integrin α5 protein expression level is associated with poor prognosis in GBM and confirms its potential as a therapeutic target implicated in the Stupp protocol resistance.

## 1. Introduction

Advances in molecular characterization technologies allowed a better definition of biomarkers, which led to the 2016 update of the World Health Organization (WHO) classification of Tumors of the Central Nervous System (CNS) integrating both histological and molecular information. Glioblastoma (GBM), a grade IV diffuse glioma, belongs to the most refractory tumors to conventional or targeted therapies [1]. Histological hallmarks of GBM include frequent mitosis, pseudo-palisades, necrosis and neoangiogenesis [2]. At the level of molecular biomarkers, GBMs are defined first by an IDH1 wild-type status. In these tumors, a marked overall genomic profile heterogeneity led to the definition of four molecular subclasses, mesenchymal, pro-neural, neural and classical forms of GBM, with the mesenchymal one being the most aggressive [3]. The prognosis of GBM patients remains very poor and has been mainly improved by the application of the Stupp protocol (temozolomide-based concomitant radiochemotherapy followed by temozolomide adjuvant therapy), which has become the standard of care [4,5], and more recently by tumor treating fields (TTFields)/Optune, which is registered in the EU and the US [6], together with likely optimization of supportive care. Numerous clinical trials targeting aberrant GBM oncogenic signaling pathways have been mostly unsuccessful, and patient survival remains short (under 20 months) [7]. New treatments are urgently required, and the definition of GBM subpopulations is supposed to be better responders to the targeting of specific pathways.

Integrins are αβ heterodimeric transmembrane proteins connecting environmental cues with cell behavior. They have remarkable dynamic properties and exist in multiple interconvertible forms, with remodeling in response to extracellular matrix (ECM) changes [8]. Integrin-mediated microenvironment sensing enables cells to adapt to stress situations by modulating cell adhesion, proliferation, survival, migration and differentiation. Integrins were initially thought to only be involved in adhesion, but they are now recognized as signaling receptors involved in numerous intracellular pathways. These pathways and the corresponding cell outcomes are critically deregulated in cancer cells. Integrin and ECM expression patterns can be highly altered in cancer cells, and integrins have been shown to strongly contribute to tumor growth and resistance to treatment [9,10,11]. Although expressed on tumor cells, several integrins are also overexpressed in tumor neovessels but poorly expressed in surrounding normal vessels, supporting a role as anti-angiogenic targets [12]. Many preclinical data support the ability of integrin antagonists to disrupt integrin signaling pathways, allowing the inhibition of angiogenesis and/or tumor growth and sensitization to treatments. The family of RGD integrins (recognizing the arginine–glycine–aspartate (RGD) motif of fibronectin, vitronectin, osteopontin etc.) is the most studied in cancer [13] and comprises among others αvβ3/β5/β6/β8 and α5β1 integrins. Integrin αvβ3 was the lead target in GBM due to its preclinically supported implications in tumor growth and neoangiogenesis. Its antagonist cilengitide was the first integrin inhibitor reaching a phase III clinical trial, called CENTRIC, but failed to show any improvement in progression-free survival (PFS) and overall survival (OS) in a patient population selected on the basis of a methylated MGMT (O-6-methylguanine-DNA methyltransferase) promoter, the well-established predictive biomarker of temozolomide [14]. A summary of integrin expression in GBM has recently been proposed [15], which includes the relationships between The Cancer Genome Atlas (TCGA) Affymetrix mRNA data and patient OS. Importantly, a group of 184 patients with newly diagnosed primary GBM treated with the standard temozolomide-based radiochemotherapy (Stupp protocol) was included in the study. The results of this analysis pointed out the impact of mRNA overexpression of ITGB1, ITGB3, ITGA5, ITGAV and ITGA3 integrin genes on the patient OS decrease, confirming previous results [10,16,17,18]. In addition, another study identified, in the same patient population, ITGB8 having similar characteristics [19].

Even if those evaluations at the genomic level attribute a role to integrins as therapeutic targets in GBM, very sparse characterizations of these integrin expressions at the protein level can be found in the literature. Several decades ago, αvβ3 integrins and their preferred ligand vitronectin were proposed to be overexpressed in GBM tissues but in small cohorts of patients, with 5 to 12 tumors of grade IV compared to about 10 tumors of grade II or III [16,20,21]. Then, works on human glioma explants [22] and patient paraffin-embedded specimens [23] proposed that α5β1, αvβ5 and β8 integrins were the predominantly over-expressed subtypes, while αvβ3 appeared in a much lower number of cases and cells [24]. Interestingly, expression analysis using rabbit monoclonal antibodies against αv-series integrins [25] revealed that although αvβ3, αvβ5 and αvβ8 integrin proteins are all overexpressed in GBM relative to the lower grades, only the αvβ3 integrin had negative prognostic significance in a cohort of about 50 patients [26], which was confirmed in another study [27]. More recently, α3 and β5 integrin protein expression were associated with tumor grade and patient survival, respectively, in a cohort of 68 [18] and 78 [28] WHO grade III and IV patients.

Although the integrin α5β1 has been suggested to be implicated in GBM aggressiveness and treatment resistance through preclinical studies [17,29] and evaluation of the genomic data bank [17,30], no data are available to confirm this hypothesis at the protein level. In glioma cell lines, we demonstrated that α5β1 integrin antagonists not only inhibit cell migration but also induce sensitization to chemotherapies. This occurs in part by alleviating the inhibition caused by the α5β1 integrin on p53 pathways [17].

In order to confirm the impact of α5β1 integrin overexpression on the clinical outcome of GBM patients, we conducted an analysis by immunofluorescence of the expression of the α5 subunit (which only associates with the β1 subunit) in a multi-center cohort of 95 patients and correlated the data with the clinical parameters of those patients. Importantly, only the patients for whom treatment with the standard protocol (Stupp protocol [4,5]) was planned were included in this study. We found that an overexpression at the protein level of this integrin was statistically associated with a worse PFS and OS as it was the case for its mRNA evaluation in the TCGA data bank. Furthermore, the clinical impact of integrin overexpression was even more significant if patients were able to receive the full course of temozolomide treatment under the Stupp protocol.

## 2. Results

### 2.1. Patient Demographics and Tumor Information

**Figure 1:**
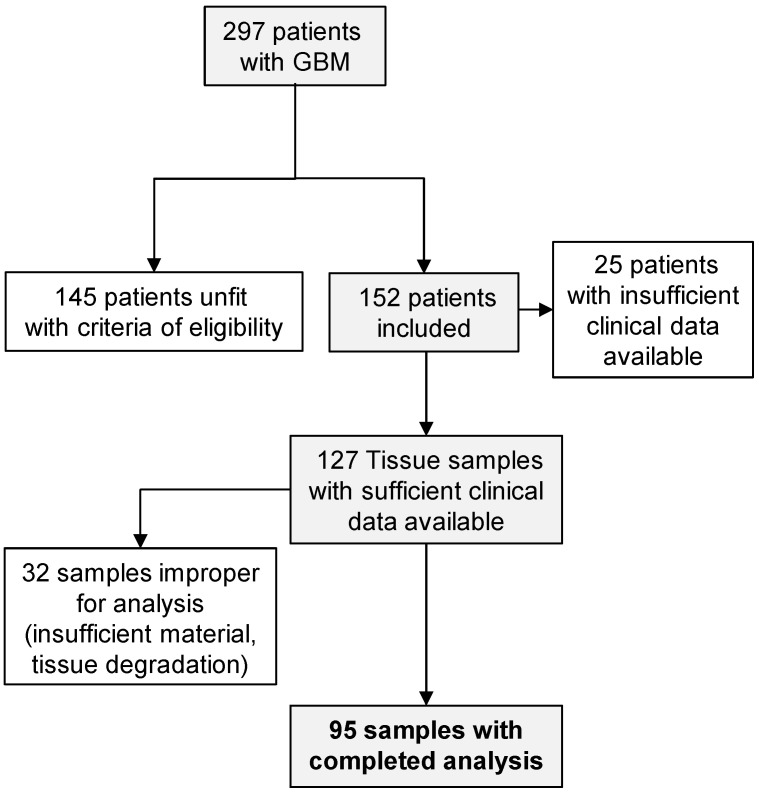
Schematic representation of tumor selection for immunohistofluorescence analysis. Glioblastoma (GBM); inclusion criteria: age between 18 and 70 years, primary glioblastoma and initially programmed for the standard Stupp protocol treatment; exclusion criteria: enrolled in clinical trial and non-standard treatment, including bevacizumab extensively off-label used in the study period in France.

To quantitatively assess integrin α5 expression in glioblastoma using immunofluorescence, a large retrospective cohort of glioblastoma tissues was obtained and annotated with demographic, molecular, clinical and follow-up information (Table 1). A total of 95 patients, consisting of 34 women (36%) and 61 men (64%) with a median age of 58 years at the time of diagnosis (minimum age, 26 years; maximum age, 70 years), were evaluated in this study (see Figure 1 for patient selection). MGMT promoter (methylated or un-methylated) and P53 status were investigated when the appropriate material was available at the time of diagnosis. Only 18 tumors were usable for the detection of the P53 antigen, indicating gene mutation in 13 cases when more than 10% of cells were positively stained by immunohistochemistry (standard procedure of hospital pathology laboratory). Tumor MGMT promoter methylation status was obtained from the medical records of 59 patients, showing that almost half of these tumors displayed an un-methylated MGMT promoter (28/59).

### 2.2. Integrin α5 Protein Expression

**Figure 2:**
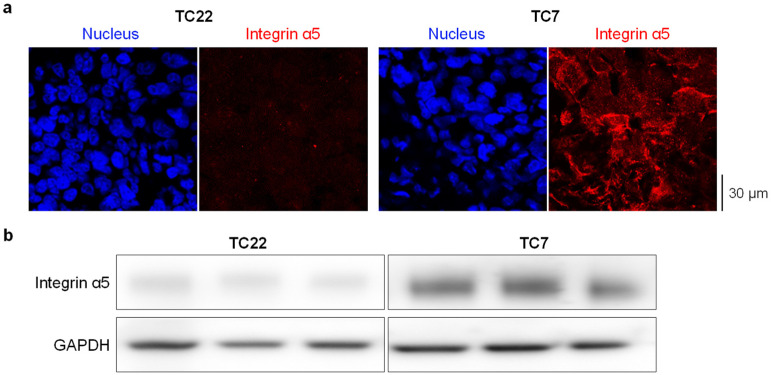
Immunofluorescence (**a**) and Western blot (**b**) on GBM-PDX tumor TC7 and TC22 presenting high and low levels of α5 integrin, respectively. In immunofluorescence, detection of integrin α5 (in red) was realized with AB1928 antibody followed by a secondary antibody coupled to Alexa Fluor^®^ 647. DAPI staining is shown in blue. One representative image per condition is shown (magnification ×63). In Western blot, detection of integrin α5 was realized in 3 xenografts from 3 different mice with H104 antibody. Anti-GAPDH antibody was used as a loading control antibody.

**Figure 3:**
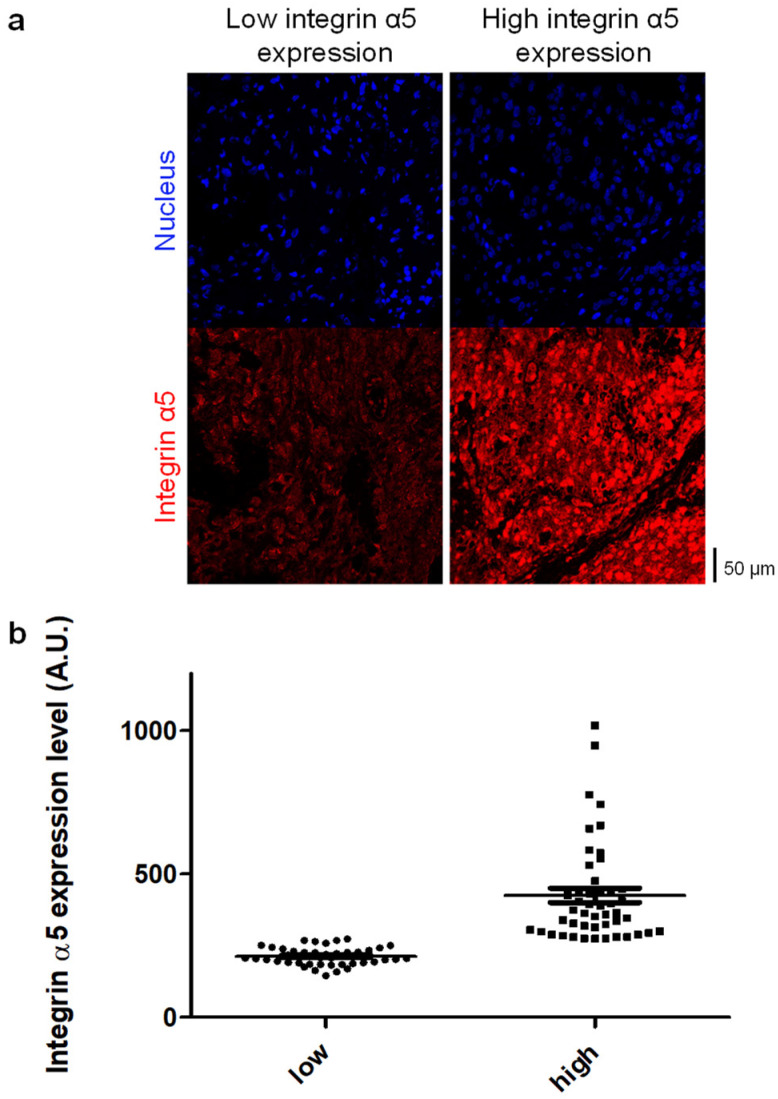
Quantification of integrin α5 protein expression level in glioblastoma. (**a**) Representative cases of low and high integrin α5 immunostaining (magnification ×40) and (**b**) distribution of cumulative data for integrin α5 expression level (MMFI expressed as arbitrary units/A.U.) in glioblastoma samples. The median of MMFI of the all cohort (275 A.U.) was used as a cut-off to distinguish 2 groups characterized by low and high integrin α5 expression levels.

Immunohistofluorescence staining of each paraffin-embedded tumor sample was applied to detect the integrin α5 protein by using AB1928 as the primary antibody. AB1928 antibody specificity (positive control) was assessed by the immunostaining of GBM-PDX tissues expressing high (TC7) and low (TC22) levels of the α5 integrin (Figure 2a) as shown previously [31]. The differential expression level of the α5 integrin in TC7 and TC22 xenografts was confirmed by Western blot analysis (Figure 2b). Secondary antibody specificity was checked by the very low fluorescence intensity (mean of mean fluorescence intensity, MMFI = 28 ± 5 A.U. from three independent tumors) observed in the immunostaining of the tumor sample in absence of the primary antibody (negative control). Nuclei counterstaining with DAPI allowed us to select several fields per tumor with homogenous tissue distribution for further analysis (Figure 3a). The mean fluorescence intensity (MFI), which takes advantage of the independent assessment of the expression level from a pathologist reading, was determined for each sample. Interestingly, the median coefficient of variation for MFI is 44% (min 12%; max 103%), showing strong intra-tumoral heterogeneity in α5 integrin expression in GBM. To observe the relationship between integrin α5 expression and patient outcome in a similar manner to that used for immunohistochemical data, but also in a rigorous manner for continuous data, we needed to define the optimal cut-off threshold. In absence of a clear overexpression of integrin α5 in a subpopulation, we decided to use the median of MMFI of the all cohort (275 A.U.) as a cut-off to distinguish two groups characterized by low (MMFI = 213 ± 4 A.U.) and high (MMFI = 425 ± 28 A.U.) integrin α5 expression levels (Figure 3b). A Mann–Whitney test indicated that MMFI regarding integrin α5 protein expression is statistically significantly different (*p* < 0.0001) between integrin α5 low- and high-expression groups. Representative images of the MMFI of both subpopulations are presented in Figure 3a. As indicated in Table 1, patient demographics (age< or >60, gender) and molecular characteristics of the tumor (MGMT promoter and P53 gene status), as well as resection degree and RPA prognostic factor, are not differently distributed (*p* > 0.05) between both subpopulations with low and high integrin α5 protein expression levels.

### 2.3. Treatment

Surgical and medical treatments are summarized in Table 1. Most of the patients underwent surgical resection of their lesion, either macroscopic (43%) or partial (36%). For the remaining patients (21%), when a surgical procedure was not indicated, tumor material was obtained by biopsy only. All the patients were selected according to inclusion criteria, including the intention of treatment by temozolomide-based radiochemotherapy followed by temozolomide adjuvant treatment (Stupp protocol) as a first line of treatment following surgery or biopsy. However, in the overall cohort, the Stupp protocol was completed in full in only 58 patients (61%) and partially completed in 24 others (25%), and, for the remaining patients, first-line treatment was either unknown (9 patients) or absent (4 patients), as indicated in the medical records. Although recurrence of the disease is almost inevitable, there is no consensus for treatment of recurrent GBM. Thirty-five patients did not receive any further treatment at recurrence (only supportive care), and data were missing for 20 other patients. The remaining patients received chemotherapy (32%) and/or bevacizumab (24%) as the second line of treatment. There were no statistically significant differences observed between the two groups (with low and high integrin α5 expression levels) according to the extent of surgery (*p* = 0.1410), the completion of the Stupp protocol (*p* = 0.4670) or the treatment choice at recurrence (*p* = 0.2103, Table 1).

### 2.4. Survival

**Figure 4:**
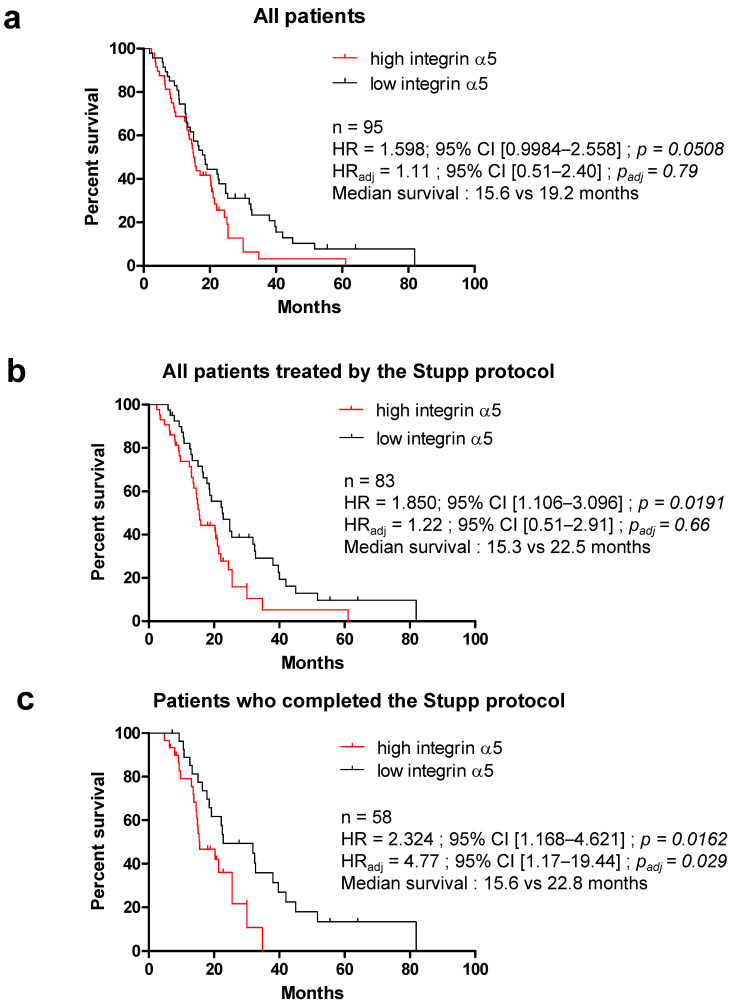
Overall survival (OS) estimated according to the expression level of integrin α5 and the completion of the Stupp protocol. Kaplan–Meier survival analysis for high and low expression levels of integrin α5 in the all cohort (**a**) in patients treated by the Stupp protocol (**b**) and in patients who completed the Stupp protocol (**c**); HR: hazard ratio in univariate analysis; HR_adj_: adjusted hazard ratio in multivariate analysis; CI: confidence interval. Survivals were compared using the log-rank (Mantel–Cox) test. *p*-values < 0.05 were considered as significant.

With a mean follow-up period of 19.6 months (95% CI, 16.7–22.5) for the 95 patients analyzed in this study, the median progression-free survival (PFS) time was 10.1 months and the median overall survival (OS) time was 17.8 months. To assess the correlation between integrin α5 protein expression and the clinical characteristics in GBM, we performed univariate and multivariate Cox regression analyses. Table 2 and Table 3 present the PFS and OS results based on the Cox proportional hazard model. P53 gene status, available only for a few patients, did not show any difference in survival. Differences in PFS were found in the univariate analysis for age (worst prognosis if >60 years old in the 76 patients analyzed, HR = 1.827, 95% CI 1.040–3.210, *p* = 0.0359); for MGMT promoter status when these data were available (*n* = 47, lower PFS if un-methylated, HR = 2.194, 95% CI 1.135–4.243, *p* = 0.0195); and for integrin α5 protein expression level, with high expression associated with a high risk of recurrence in the all cohort (HR = 1.696, 95% CI 1.031–2.792, *p* = 0.0377). The extent of surgical resection only had a significant impact on OS (*p* < 0.001) and not PFS (*p* = 0.3289), with better prognosis associated with partial or macroscopic resection versus biopsy. The same results were obtained with RPA class < V (recursive partitioning analysis, taking into account age, surgery extent and Karnofsky performance status; *p* = 0.1596 for PFS and *p* = 0.0120 for OS). The benefit of a methylated MGMT promoter in the tumor for patient survival was also observed in OS when data were available (59/95 patients, HR = 2.217 un-methylated vs. methylated, 95% CI 1.031–2.792, *p* = 0.0377). A high integrin α5 protein expression level was associated with a decreased OS in the all cohort (median survival: 15.6 vs. 19.2 months, Figure 4a) but not significantly (*p* = 0.0508). However, univariate subgroup analysis based on first-line treatment indicated that the difference in survival based on integrin α5 protein expression was even more significant if the Stupp protocol [4,5] was completed (temozolomide-based radiochemotherapy plus temozolomide adjuvant treatment, median survival: 15.6 vs. 22.8 months; HR = 2.324; 95% CI 1.168–4.621, *p* = 0.0162, Figure 4c). All variables with *p* < 0.05 observed in the univariate survival analysis (age, resection degree, RPA, MGMT promoter status and integrin α5 protein expression level) were included in the multivariate analysis. Methylation of the MGMT promoter was the only significant prognostic factor for PFS in both the all cohort (HR = 4.71; 95% CI 1.43–8.93, *p* = 0.0065) and the subgroup of patients treated by the Stupp protocol (completed or not, HR = 4.46; 95% CI 1.68–11.84, *p* = 0.0027). The benefit of MGMT promoter methylation was confirmed for OS in every patient and even more significantly in Stupp-treated patients (median survival: 15.6 vs. 25.7 months, HR = 6.95; 95% CI 2.45–19.72, *p* = 0.0002). In the multivariate analysis, a high expression level for integrin α5 was identified as an independent prognostic factor for OS but only in the subpopulation of patients who completed the standard first-line treatment, the Stupp protocol (HR = 4.77; 95% CI 1.17–19.44, *p* = 0.029, Figure 4c).

## 3. Discussion

Several factors are attributed to GBM prognosis, depending on clinical and biological patient parameters, such as age and extent of resection, or based on the characteristics of the tumor at the molecular level, such as IDH mutation or MGMT promoter methylation. According to our results and the data available, the cohort we examined was consistent with previous results [32,33,34].

Although several integrins are described as pertinent therapeutic targets in GBM, the first clinical trial with an αvβ3 integrin antagonist failed [14,35]. Other integrins may be at the forefront for fighting GBM, but their real implication in the clinical outcome of patients has to be confirmed before the proposal of new targeted therapies. We and others proposed the α5β1 integrin as an additional player in the aggressiveness of GBM and showed that a high level of ITGA5 mRNA correlated with a worse outcome of patients [17,30]. Our previous preclinical results also pointed to its role in tumor growth and resistance to glioma therapies [17,36]. We show here for the first time that the protein overexpression of the α5 integrin subunit in the tumor cells of GBM is linked to worse PFS and OS of patients, especially those submitted to the standard Stupp protocol. This result is the first step to confirm that targeting the α5β1 integrin may be of interest in the field of GBM.

Other arguments are already available based on clinical sample investigations. The ITGA5 gene is found in the signature of the mesenchymal subclass of GBM [3,15], considered as the most aggressive and resistant phenotype until now. Hence, single cell-derived clonal analysis described a drug and radiation resistance phenotype for cells overexpressing the α5β1 integrin [37,38]. In fact, we observed in our series of samples that some of them had a high index of intra-tumoral heterogeneity with areas of intense staining close to unstained regions, as indicated by a high coefficient of variation of Mean Fluorescence Intensity (MFI). The fluorescent staining intensity used for the present study consists of a mean of the different area intensities, which may underestimate the final analysis. Spatial (perivascular vs. perinecrotic niches, tumor core vs. tumor edge) and temporal (primary and recurrent tumors) molecular and functional heterogeneity of a given tumor is nowadays acknowledged to be part of clinical trial failures [39]. Obviously, intra- or inter-tumoral heterogeneity of the α5β1 integrin or other integrin expression may have implications in the failure of targeted therapies in unselected populations of patients.

As an example, in the CENTRIC phase III clinical trial, cilengitide (an αvβ3/β5 specific inhibitor), administered in combination with the Stupp protocol in GBM with a methylated MGMT promoter, failed to show improved outcomes, although previous phase II studies had encouraging results [14]. Similarly, the CORE phase II clinical trial, with cilengitide in patients with unmethylated MGMT GBM, showed negative results [35]. Interestingly, evaluation of the αvβ3, αvβ5 and αvβ8 integrin expressions in the tumor tissues from both trials led to the conclusion that only overexpression of αvβ3 in tumor cells (and not in the endothelial cells) in the CORE trial was predictive of the beneficial effect of cilengitide [40]. Moreover, a recent elegant study revealed that the overexpression of the β3 integrin subunit in GBM may be coupled with an addiction of the tumor to glucose through Glut3 to render the tumor sensitive to cilengitide [41]. Whether overexpression of the α5β1 integrin is coupled to other signaling pathways that may be targeted simultaneously remains to be determined. Work is in progress in our laboratory to determine synthetic lethality partners of this integrin, but data from the literature can already point to some examples [42,43,44,45,46].

Glioblastoma oncogenesis has long been linked to epidermal growth factor receptor (EGFR) gene amplification, mutations and constitutive activation. Nevertheless, nimotuzumab targeting EGFR in a phase III clinical trial (NCT00753246) proved unable to improve PFS and OS when administered with the standard Stupp protocol [47]. Interestingly, crosstalk between integrins and growth factor receptors, including EGFR, has been largely described in several solid tumors, including glioma [42]. Cooperation between β1 integrins and EGFR led to resistance to EGFR-targeted therapies through different molecular pathways [42,43]. It is thus tempting to propose that the α5β1 integrin and EGFR inhibitors may be included in a combination protocol for selected patients.

Angiogenesis is a key feature of GBM, and its associated signaling pathways rapidly became potential targets for therapy [48]. The αvβ3 integrin was described as a marker of tumor neoangiogenesis and cilengitide proposed as an anti-angiogenic agent [49]. This concept was controversial [50], and overexpression of the αvβ3 integrin on tumor vessels did not correlate with the improved effects of cilengitide [14,35]. Conversely, the α5β1 integrin behaves as such a marker without ambiguity [51,52]. Unfortunately, no clinical data are available to check the anti-angiogenic effects of α5β1 inhibitors. The prominent molecule activating tumor angiogenesis is VEGF [53]. Bevacizumab, targeting circulating VEGF, was evaluated in two phase III clinical trials, AVAGLIO and RTOG-0825. Both trials gave similar results, showing an improvement in PFS not accompanied by an improvement in OS [54,55]. Recently, analysis of the OS of patients treated with bevacizumab in the GBM-IDH wild-type TCGA cohort indicated a significant improvement when VEGFA was overexpressed [56]. Unfortunately, the response to bevacizumab is only transient and recurrence due to resistance occurs [48]. Microarray transcriptional analysis of paired naïve and recurrent tumors revealed two different populations of resistant tumors. One of them exhibited enhanced expression of the α5 integrin and its ligand fibronectin [44]. In line with this, targeting the β1 integrin (with which α5 integrin dimerizes) has been proposed to circumvent resistance to bevacizumab in GBM [45], either as a monotherapy for naïve tumors naturally resistant to bevacizumab or combined with bevacizumab to reduce the risk of acquired resistance [46]. From this last example, our immunological analysis of α5 integrin expression may be the basis of a combined bevacizumab–α5 integrin inhibitor proposal for tumor patients highly expressing VEGF/α5 integrin.

Heterogeneity of GBM at the molecular level highlights the need for patient selection to identify a subgroup of patients with true target-specific dependency [3]. Interestingly, post hoc evaluations of subgroups in the above trials tend to confirm that evaluation of not only the target expression at the protein level but also its associated or cross-reacting signaling pathways must be taken into consideration [40,44]. Concerning the α5β1 integrin in GBM, some lines of approach may be considered for future investigations. Firstly, it may be suggested that a prominent role of this integrin will be found in mesenchymal GBM inasmuch as probes of a correlation between mRNA and protein levels will be given in this subclass of tumors; secondly, extrapolation from our preclinical results on the negative relationships between the integrin and p53 signaling may indicate that blocking the integrin in a p53 WT background in addition with reactivation of p53 signaling (as for example with mdm2 inhibitors) will be more effective [17,36]. As shown in the examples above, other combined therapies may be useful to fight GBM inasmuch as partners are identified on the targeted tumors.

Our results support the integrin α5β1 as a pertinent target in GBM to circumvent standard Stupp protocol resistance, but further work is needed before proposing clinical trials in order to determine if a selected subpopulation of patients may be suitable for them. Interestingly, phase II clinical trials have already been carried out with specific anti-α5β1 integrin inhibitors, such as volociximab [57] and MINT1526A [58], two antibodies that block the interaction between the integrin and its extracellular matrix ligands. Both trials reported safe administration and an absence of adverse events as monotherapies in patients with advanced solid tumors, but clinical benefit was still insufficient. In these trials, GBM was not included. Such drugs may be proposed as targeted therapies in selected high α5 integrin expressers based on our results.

## 4. Materials and Methods

### 4.1. Patients and Tissue Samples

We retrospectively screened a total of 297 patients with glioblastoma treated by radiotherapy in five different referral centers (University Hospital Center from Amiens and four local Hospital Centers from Compiègne, Compiègne-Creil, Saint Quentin and Beauvais) between January 2006 and December 2013. Among these, 145 cases were excluded according to inclusion criteria (age between 18 and 70 years, primary glioblastoma and initially programmed for the standard Stupp protocol treatment) and exclusion criteria (enrolled in a clinical trial and non-standard treatment, including bevacizumab extensively off-label used in the study period in France). An additional 57 cases were excluded due to non-complete medical records or unavailable or insufficient tumor material for immunofluorescence analysis (Figure 1).

For each of the 95 selected patients, the following clinico-histopathological data were recorded and anonymized prior to analysis: gender, age, molecular biology (p53 and MGMT status) when available, type of surgery (macroscopic, subtotal resection or biopsy), adjuvant treatment and treatment at recurrence. RPA (recursive partitioning analysis) score was also calculated. Complete patient clinical data are reported in Table 1. The medical records of all patients included were checked up to 20 June 2015 to determine the follow-up period. All patients were asked at the time of diagnosis to give their consent for using a portion of the tumor tissue sample initially obtained for diagnosis, not needed for further clinical use but for research or potential future research. At the time of study, alive patients were additionally asked to confirm their consent to the study, and the absence of any opposition for other patients was checked. The corresponding ethical consent is available in the University Hospital Center from Amiens, France. The study protocol was approved by a local French ethical committee for the evaluation of non-interventional research, a sub-commission of the committee for the protection of individuals, on 10 December 2015.

### 4.2. Patient-Derived Xenografts

Two patient-derived ectopic xenografts (PDXs) were used to establish antibody specificity [29]. TC7 and TC22 GBM-PDXs expressing high and low levels of the α5 integrin, respectively, were produced, paraffin-embedded and mounted on glass slides as previously described [17,59].

### 4.3. Immunohistofluorescence

All tissues (biopsies, excised tumors) were provided by the Pathology Department of the University Hospital Center from Amiens as formalin-fixed paraffin-embedded (FFPE) tumor sections (5 µm thickness). TC7 and TC22 GBM-PDXs were also used as FFPE tumor sections. Both human tumors and GBM-PDX FFPE sections were deparaffinized, rehydrated and subjected to unmasking antigen protocol using Dako retrieval solution (Tris/EDTA) pH9 (Agilent Technologies, Les Ulis, France). Next, blocking buffer (5% goat serum, 0.1% Tween-20, PBS) was applied for 1 h at room temperature. The integrin α5 protein was labeled with rabbit antibody directed against the α5 integrin cytoplasmic tail (AB1928, 1/300, Merck-Millipore, Molsheim, France) by overnight incubation at 4 °C. After washing in PBS-Tween 0.1%, tissue sections were incubated for 1 h with the appropriate secondary antibody (goat anti-rabbit IgG Alexa Fluor^®^ 647, 1/200, Invitrogen—ThermoFischer, Coutaboeuf, France). Nuclei were counterstained with DAPI (10 mg/mL, Sigma-Aldrich, St Quentin Fallavier, France), and coverslips were mounted onto tissue section using Permafluor Aqueous Mounting Medium (Fisher Scientific, Illkirch, France). Images were acquired using a confocal laser scanning microscope Leica TCS SPEII at 40× magnification (objective ×20 N.A. 0,7, HCX PL APO CS; zoom ×2) from the PIQ Quantum Efficiency Strasbourg platform (http://quest.igbmc.fr/, accessed on 2 July 2021). The following settings were applied for DAPI and Alexa Fluor^®^ 647, respectively: DAPI: excitation wavelengths = 405 nm, laser power of 15%, emission spectrum = 430–480 nm, gain = 630 volts, off-set = −0.36; Alexa Fluor^®^ 647: excitation wavelengths = 635 nm, laser power of 19.5%, emission spectrum = 647–740 nm, gain = 830 volts, off-set = −0.53. The speed of the scanner was 400 Hz (512 × 512 pixels). All confocal microscope settings were maintained constant between each staining experiment. Approximatively 10 (6 to 20) different fields per slide were randomly selected according to both the size of the tissue section (low amount of tissue in the case of biopsies) and the heterogeneity of the tumor tissue (more images captured if staining was heterogeneous). The abundance of the integrin α5 protein was estimated as the mean red fluorescence intensity (MFI) per field by using ImageJ software version 1.50 [60], and the mean of MFI (MMFI) was calculated for each tumor.

### 4.4. Western Blot

Frozen TC22 and TC7 GBM-PDX tissues from 3 different mice were ground in liquid nitrogen and lysed in RIPA buffer. Proteins were then extracted and analyzed by Western blot as previously described [17,31]. Briefly, 10 μg of protein was separated by SDS-PAGE (Bio-Rad, Schiltigheim, France) and transferred to polyvinylidene fluoride (PVDF) membranes (Bio-Rad, Schiltigheim, France). Blots were probed with antibodies to α5 integrin (H104, Santa Cruz Biotechnology, Heidelberg, Germany) and to glyceraldehyde 3-phosphate dehydrogenase (GAPDH; Merck-Millipore, Molsheim, France), the latter used as the loading control for the tissue lysate samples. Horseradish peroxidase (HRP)-coupled secondary antibody from Promega (Charbonnières, France) allowed us, through enhanced chemiluminescence, to visualize the protein with the LAS4000 imager.

### 4.5. Statistical Analysis

We used the mean (± standard error of the mean, SEM) values and frequencies (percentages) for the description of continuous and categorical variables, respectively. Means and proportions were compared using the Mann–Whitney test and the chi-squared test (or Fisher’s exact test, if appropriate), respectively. Progression-free survival (PFS) was estimated from the date of surgery to the date of the first recurrence. Overall survival (OS) was defined as the time between the date of surgery and the date of the last follow-up or the date of death from any cause. If no event was observed, patients were censored at the last follow-up. Survival curves were built using the Kaplan–Meier method and compared using the log-rank test. Survival was described by median with 95% confidence interval (CI) for PFS and OS. Hazard ratios were used to calculate the relative risk of death or progression. All variables with *p* < 0.05 observed in the univariate survival analysis were included in a multivariate Cox regression model with stepwise backward elimination to estimate HR with a 95% CI and to select potential prognostic factors. *p*-values < 0.05 were considered as significant. Analyses were performed with GraphPad Prism software version 5.04 and R software version 3.6.0.

## 5. Conclusions

Overall, our study confirms that overexpression of the α5 integrin subunit in GBM is associated with worse survival, especially in patients undergoing the standard Stupp protocol, strengthening the argument for a role of this integrin in resistance to temozolomide and/or radiotherapy. Integrin α5β1 thus remains a promising therapeutic target for GBM treatment, and optimized preclinical and clinical research is mandatory to identify and validate new drug candidates.

## Figures and Tables

**Table 1:** Clinicopathological features of the 95 GBM (glioblastoma) patients, comparing low integrin α5 and high integrin α5 groups according to median expression level (median of MMFI) as the cut-off threshold.

Clinicopathological Features	Total No. of Patients	With	With	
		Low α5	High α5	*p*-Value *
		Expression	Expression	
n	95	47	48	
Age, n (*%*)				
≥60	36 (*38*)	19 (*40*)	17 (*35*)	*0.6752*
<60	59 (*62*)	28 (*60*)	31 (*65*)	
Gender, n (*%*)				
Male	61 (*64*)	26 (*55*)	35 (*73*)	*0.0891*
Female	34 (*36*)	21 (*45*)	13 (*27*)	
Resection degree, n (*%*)				
Biopsy	20 (*21*)	6 (*13*)	14 (*29*)	
Partial resection	34 (*36*)	18 (*38*)	16 (*33*)	*0.1410*
Macroscopic resection	41 (*43*)	23 (*49*)	18 (*38*)	
RPA score, n (*%*)				
<V	37 (*39*)	17 (*36*)	20 (*42*)	
≥V	37 (*39*)	19 (*40,5*)	18 (*37*)	*0.8575*
Unknown	21 (*22*)	11 (*23,5*)	10 (*21*)	
MGMT promoter status, n (*%*)				
Methylated	31 (*33*)	17 (*36*)	14 (*29*)	
Un-methylated	28 (*29*)	14 (*30*)	14 (*29*)	*0.6962*
Unknown	36 (*38*)	16 (*34*)	20 (*42*)	
P53 gene status, n (%)				
Mutated (antigen detected in >10% cells)	13 (*14*)	3 (*6*)	10 (*21*)	
Wild-type (<10% cells)	5 (*5*)	2 (*4*)	3 (*6*)	*0.0912*
Unknown	77 (*81*)	42 (*90*)	35 (*73*)	
Stupp protocol, n (%)				
Completed	58 (*61*)	28 (*60*)	30 (*63*)	
Un-completed	24 (*25,5*)	12 *(25)*	13 (*27*)	*0.4670*
None	4 *(4)*	1 *(2)*	2 (*4*)	
Unknown	9 (*9,5*)	6 (*13*)	3 (*6*)	
Recurrence treatment, n (%)				
None	35 (37)	14 (30)	21 (44)	
Second-line treatment	40 (42)	19 (42)	20 (42)	
-Chemotherapy	30 (32)	14 (30)	16 (33)	*0.2103*
-Bevacizumab	23 (24)	9 (19)	14 (29)	
Unknown or NA	20 (21)	14 (30)	7 (15)	
α5 expression level (A.U.) Mean ± SE	320 ± 17	213 ± 4	425 ± 28	*p* < 0.0001

MMFI: Mean of mean fluorescence intensity; RPA: recursive partitioning analysis; MGMT: O-6-methylguanine-DNA methyltransferase; TMZ: temozolomide; BEV: bevacizumab; AU: arbitrary unit; NA: not applicable; SE: standard error *. Chi-square test was used to evaluate independency between clinico-pathological features and integrin α5 expression level, and Mann–Whitney test was used to compare expression level (MMFI) of integrin α5 between low- and high-expression subgroups.

**Table 2:** Univariate analysis of factors associated with survival and progression.

Variables	PFS				OS			
	*n*	HR	95% CI	*p* *	*n*	HR	95% CI	*p* *
**Age**								
≥60 years vs. <60 years	76	1.827	1.040–3.210	0.0359	*95*	1.989	1.195–3.308	*0.0093*
**Resection degree**	76			0.3289	*95*			*<0.0001*
Biopsy vs. Partial	39	1.521	0.646–3.579	0.3367	*54*	7.764	3.344–18.170	*<0.0001*
Biopsy vs. Macroscopic	47	2.113	0.848–5.265	0.1084	*61*	9.925	3.900–21.820	*<0.0001*
Partial vs. Macroscopic	66	1.150	0.682–1.940	0.5995	*75*	1	0.598–1.672	*0.9996*
**RPA**	76			0.1596	*95*			*0.0120*
≥V vs. <V	59	1.560	0.868–2.803	0.1367	*74*	2.106	1.230–3.607	*0.0066*
**P53 gene status**	76			0.7537	*95*			*0.8356*
Mutated vs. Non Mutated	15	0.597	0.151–2.360	0.4622	*18*	0.597	0.128–2.784	*0.5118*
**MGMT promoter status**	76			0.0834	*95*			*0.0690*
Un-Methylated vs. Methylated (All)	47	2.194	1.135–4.243	0.0195	*59*	2.217	1.184–4.151	*0.0129*
Un-Methylated vs. Methylated (Stupp)	45	2.547	1.292–5.021	0.0069	*53*	2.497	1.278–4.880	*0.0074*
Un-Methylated vs. Methylated (Completed Stupp)	31	2.083	0.926–4.682	0.0759	*35*	2.062	0.872–4.872	*0.0992*
**Integrin α5**								
High vs. Low expression (All)	76	1.696	1.031–2.792	0.0377	*95*	1.598	0.998–2.558	*0.0508*
High vs. Low expression (Stupp)	72	1.771	1.055–2.975	0.0307	*83*	1.805	1.106–3.096	*0.0191*
High vs. Low expression (Completed Stupp)	53	1.635	0.879–3.040	0.1206	*58*	2.324	1.168–4.621	*0.0162*

PFS: progression-free survival; OS: overall survival; HR: hazard ratio; 95% CI: 95% confidence interval; y: year; RPA: recursive partitioning analysis; MGMT: O-6-methylguanine-DNA methyltransferase. * log-rank test, *p* < 0.05 was considered as significant.

**Table 3:** Multivariate Cox regression analysis for survival.

Variables	PFS			OS		
	HR	95% CI	*p* *	HR	95% CI	*p* *
**All patients**						
Age (≥60 years vs. <60 years)	1.38	0.51–3.72	0.52	1.35	0.54–3.33	*0.52*
Resection degree (low vs. high)	2.03	0.95–4.35	0.0683	3.72	1.60–8.64	*0.0023*
RPA (≥V vs. <V)	1.97	0.80–4.86	0.14	1.43	0.61–3.35	*0.42*
MGMT (Un-Methylated vs. Methylated)	4.71	1.43–8.93	0.0065	4.71	1.85–11.90	*0.0011*
Integrin α5 (high vs. low)	1.28	0.57–2.88	0.55	1.11	0.51–2.40	*0.79*
**Stupp**						
Age (≥60 years vs. <60 years)	1.28	0.45–3.69	0.64	1.31	0.48–3.60	*0.58*
Resection degree (low vs. high)	1.87	0.86–4.07	0.12	3.73	1.52–9.16	*0.0040*
RPA (≥V vs. <V)	1.83	0.69–4.88	0.22	1.71	0.45–3.07	*0.75*
MGMT (Un-Methylated vs. Methylated)	4.46	1.68–11.84	0.0027	6.95	2.45–19.72	*0.0002*
Integrin α5 (high vs. low)	1.28	0.55–3.01	0.57	1.22	0.51–2.91	*0.66*
**Completed Stupp**						
Age (≥60 years vs. <60 years)	2.48	0.61–10.09	0.20	2.20	0.53–9.09	*0.27*
Resection degree (low vs. high)	1.41	0.54–3.68	0.48	2.04	0.60–6.90	*0.25*
RPA (≥V vs. <V)	1.16	0.31–4.28	0.83	3.12	0.73–13.25	*0.12*
MGMT (Un-Methylated vs. Methylated)	2.58	0.76–8.76	0.128	4.27	1.08–16.83	*0.038*
Integrin α5 (high vs. low)	2.09	0.63–6.90	0.22	4.77	1.17–19.44	*0.029*

PFS: progression-free survival; OS: overall survival; HR: hazard ratio; 95% CI: 95% confidence interval; y: year; RPA: recursive partitioning analysis; MGMT: O-6-methylguanine-DNA methyltransferase. * multivariate COX regression analysis, *p* < 0.05 was considered as significant.

## Data Availability

All data are contained within the article.
